# Evidence of host specificity in *Lactobacillus johnsonii* genomes and its influence on probiotic potential in poultry

**DOI:** 10.1016/j.psj.2023.102858

**Published:** 2023-06-10

**Authors:** Abigail Johnson, Elizabeth A. Miller, Bonnie Weber, Cristian Flores Figueroa, Jeannette Munoz Aguayo, Anup Kollanoor Johny, Sally Noll, Jeanine Brannon, Briana Kozlowicz, Timothy J. Johnson

**Affiliations:** ⁎ Department of Veterinary and Biomedical Sciences, University of Minnesota, Saint Paul, MN, USA; † Mid-Central Research and Outreach Center, University of Minnesota, Willmar, MN, USA; ‡ Department of Animal Science, University of Minnesota, Saint Paul, MN, USA; § Cargill, Incorporated, Wayzata, MN, USA

**Keywords:** probiotic, host adaptation, *lactobacilli*, turkey, broiler

## Abstract

To date, the selection of candidate strains for probiotic development in production animals has been largely based upon screens for desired phenotypic traits. However, increasing evidence indicates that the use of host-specific strains may be important, because coevolution with the animal host better prepares a bacterial strain to colonize and succeed in its respective host animal species. This concept was applied to *Lactobacillus johnsonii* in commercial poultry production because of its previous correlation with enhanced bird performance. Using 204 naturally isolated chicken- and turkey-source *L. johnsonii*, we demonstrate that there is a strong phylogenetic signal for coevolution with the animal host. These isolates differ phenotypically, even within host source, and these differences can be correlated with certain *L. johnsonii* phylogenetic clades. In commercial turkey poults, turkey-specific strains with strong in vitro phenotypes performed better early in life than strains lacking those phenotypes. A follow-up performance trial in broiler chickens demonstrated that chicken-specific strains result in better overall bird performance than nonchicken-specific strains. Collectively, this work provides evidence for the impact of host adaptation on a probiotic strain's potential. Furthermore, this top-down approach is useful for screening larger numbers of isolates for probiotic candidates.

## INTRODUCTION

Modern poultry production relies on the ability to sustain high levels of performance, while maintaining optimal flock health. In reality, this simple concept is quite challenging to achieve. Poultry production is faced with numerous challenges, including necessary reductions in antimicrobial usage, our changing climate, and emergent and re-emergent diseases. To face these challenges, high quality feed and an arsenal of feed additives are essential.

One aspect of maintaining a healthy bird is managing its microbiota. Again, this is a simple concept which is enormously challenging to achieve. The gastrointestinal microbiota of a healthy bird is rich and complex yet, in many ways, fragile (Johnson et al., 2018). Modern poultry production reduces the opportunities for the acquisition of natural microflora due to procedures used to prevent pathogens from entering production systems (Dittoe et al., 2022). The use of antimicrobials and disinfectants further exacerbates the challenge by directly influencing the gastrointestinal and barn microbiota, respectively (Ae Kim et al., 2017; Ward et al., 2019; Robinson et al., 2020; Fan et al., 2022). Finding the balance between maintaining a rich and diverse microbiota, yet reducing pathogen load, often necessitates the use of feed additives.

Many feed additives exist for poultry, including the use of live beneficial microbials. These are also referred to as direct-fed microbials (**DFMs**) or probiotics. One would intuitively conclude that using any live beneficial microbial in feed will have a positive impact on the gastrointestinal microbiota. In theory, this should be true. However, a number of studies have shown that vegetative and spore-forming beneficial bacteria applied to poultry exert a modest effect on the gut microbiome, at best (Mohamed et al., 2021; Chang and Yu, 2022). These effects can also be polarizing, often with data indicating both positive and negative effects on the microbiome (Zou et al., 2018). However, there is evidence that DFMs could accelerate the maturation of the poultry gut microbiome (Ward et al., 2019; Bilal et al., 2021). A barrier with this approach is that most DFMs in poultry are suggested for continuous use in feed, or frequent application in drinking water. This can become costly over time, particularly as feed intake increases with older birds. Ideally, use of a DFM which is host-adapted could alleviate the need for continuous or frequent application through strong colonization of its appropriate host.

Previous work has explored the idea of using host-adapted microbes for DFM application in poultry, including the use of *Lactobacillus johnsonii*. Specifically, we demonstrated that a turkey-source, *L. johnsonii*-containing DFM has the potential to increase early turkey poult performance through positive modulations in the gut microbiome (Ward et al., 2019). This work indicated that host-adapted DFMs have the potential to mimic some of the positive performance and microbiome-enhancing effects exerted by antibiotics used for growth promotion. Similar work has been achieved using a chicken-adapted *L. johnsonii* strain in broilers (Wegmann et al., 2009). Previous work suggests that coevolution occurs between lactobacilli and their hosts (Buhnik-Rosenblau et al., 2012), which could play an important role in their subsequent colonization and success within different hosts. However, even within bacterial species and host animal source, choosing an appropriate strain with probiotic properties is still important. Strain-level differences exist regarding phenotypic properties such as pathogen inhibition and tolerance to gut transit conditions (Wang et al., 2022). Ideally, a DFM candidate for promotion of early life poultry performance and gut microbiome development should be both host-adapted and possess some of the positive phenotypes of what is considered a good probiotic. Here, we present an approach towards identifying and testing host-specific probiotic strains. Using host-specific *L. johnsonii* from broilers and commercial turkeys, we identified optimal probiotic candidate strains and subsequently determined if host adaptation influences probiotic efficacy in the bird.

## MATERIALS AND METHODS

### Bacterial Isolates

We focused on *L. johnsonii* because previous work had identified this species as being positively correlated with either bird-level or flock-level weights in commercial broilers and turkeys, using microbiome-based data (Johnson et al., 2018; Ward et al., 2019). Based upon this work, *lactobacilli* isolates were first collected from ileal and cecal contents of commercial and research turkey flocks. Three historically high-performing flocks were used, including 2 commercial turkey flocks and 1 research turkey flock housed in pens at the University of Minnesota (Rosemount, MN). Samples were collected at wk 1, 2, 3, 4, 5, 6, 8, and 10 of age. From each flock and time point, 5 birds were euthanized and sampled (40 birds per flock, 120 birds total). Ileal and cecal contents were aseptically collected from each bird, and 1 g of homogenate was placed in 10 mL sterile phosphate-buffered saline (**PBS**). Four 10-fold serial dilutions were performed for each sample, and 100 µL of each dilution was plated on De Man, Rogosa and Sharpe (MRS) agar (BD Difco, Franklin Lakes, NJ). Plates were placed in Mitsubishi boxes with anaerobic gas packs (AnaeroPack, Mitsubishi Gas Chemical Company, Tokyo Japan) and incubated for 24 to 48 h at 37ºC. Up to 10 isolated colonies per sample were picked and regrown in MRS broth for 24 to 48 h at 37ºC. Isolates were stored in 20% glycerol at -80ºC until further use.

For initial speciation of commercial turkey isolates, PCR was performed with F342 and 518R 16S rRNA universal primers, 5′-CCTACGGGAGGCAGCAG-3′ and 5′-ATTACCGCGGCTGCTGG-3′, following a previously described protocol (Human Microbiome Project et al., 2012). Sanger sequencing was performed on each amplicon at the University of Minnesota Genomics Center. Following sequencing, each forward and reverse read was quality trimmed and aligned using DNASTAR version 9.0 software (Lasergene, Madison, WI). Sequences were then searched against NCBI reference genomes using BLASTN for best hit analysis to assign closest matching bacterial species. PCR was then performed on samples with >95% hits to *L. johnsonii*, for *L. johnsonii* confirmation, using previously published *L. johnsonii*-specific primers (Furet et al., 2004). Primer sequences used were 5′ -CACTAGACGCATGTCTAGAG-3′ and 5′-AGTCTCTCAACTCGGCTATG-3′, producing an expected 107 bp product if positive. Each PCR run included appropriate positive and negative control DNA. PCR was performed using GoTaq Flexi (Promega, Madison, WI), and each reaction contained 5 µL buffer, 1.8 µL of 25 mM MgCl_2_, 1 µL each of 10 mM forward and reverse primer, 0.5 µL of 100 mM dNTP mix, 0.25 µL of GoTaq DNA polymerase, 2 µL of template DNA, and 13.25 µL of nuclease-free water (Qiagen, Germantown, MD). PCR conditions were 95ºC for 2 min, then 30 cycles of 95ºC for 30 s, 54ºC for 30 s, and 72ºC for 30 s. The reaction was completed with 72ºC for 7 min followed by a hold at 4ºC until agarose gel electrophoresis.

For isolation of *L. johnsonii* from commercial broilers, a single integrated company was enrolled to sample 4 different flocks aged 7, 14, 21, and 28 d. From each flock, 5 birds per flock were sampled (20 birds total). Ileum samples were aseptically collected from each bird and serially diluted in PBS. Dilutions were then plated on both MRS and *Lactobacillus* selection (**LBS**) agar (BD Difco, Franklin Lakes, NJ). Ten colonies were then picked per sample from an appropriate dilution plate displaying isolated colonies. Each colony was subsequently grown in LBS broth overnight at 37ºC with shaking. DNA was extracted from each growth using the Qiagen DNEasy Kit following the manufacturer's recommendations for Gram positive bacteria. PCR was performed on each sample to determine if an isolated colony was *L. johnsonii*, again using previously published *L. johnsonii*-specific primers (Furet et al., 2004).

### DNA Sequencing and Analysis

All broiler and turkey isolates identified as *L. johnsonii* were sequenced in this study using Illumina HiSeq or MiSeq (n = 118 turkey and n = 85 chicken). Genomic DNA libraries were created using the Nextera XT library preparation kit and Nextera XT index kit v2 (Illumina, San Diego, CA), and sequencing was performed using 2×150-bp or 2×250-bp dual-index runs on an Illumina HiSeq or MiSeq, respectively. All raw FASTQ files were trimmed and quality filtered using Trimmomatic (v0.33) (Bolger et al., 2014), removing Illumina Nextera adapters and quality filtering using a sliding window of 4 with an average Phred quality score of 20. Trimmed reads were de novo assembled using the Shovill pipeline (v1.0.4), which utilizes the SPAdes assembler (Bankevich et al., 2012), with default parameters (https://github.com/tseemann/shovill). Assembly quality was assessed with QUAST (v5.0.0) (Gurevich et al., 2013).

Additionally, 1 turkey-source isolate from this study were sequenced using PacBio to close its genome (UMNLJ21). Chemistry used was P6-C4 with a target of approximately 50x coverage per genome. Hybrid assemblies were performed using CLC Genomics Workbench Microbial Genomics Module v. 2014, with default settings.

For phylogenetic analyses, core genome single nucleotide polymorphisms (**SNPs**) were identified in each isolate's genome using Snippy (v4.4.0), with a minimum sequencing depth of 8x (https://github.com/tseemann/snippy) and using *L. johnsonii* strain UMNLJ21 as the reference. Additionally, raw reads from *L. johnsonii* isolates available in the NCBI database (n = 45) were used for comparison purposes. Core SNP alignments were then created for all isolates (n = 250). Recombinant regions were identified with Gubbins (v2.3.4) (Croucher et al., 2015) and masked from the core genome alignments using maskrc-svg (v0.5) (https://github.com/kwongj/maskrc-svg). The program snp-sites (v2.4.1) were then used to extract all core SNPs and monomorphic sites where the columns did not contain any gaps or ambiguous bases (Page et al., 2016). Pairwise core SNP distance matrices were created using snp-dists (v0.6.3) (https://github.com/tseemann/snp-dists) after duplicate core SNP profiles were removed with SeqKit (v0.10.1) (Shen et al., 2016).

Maximum likelihood trees for all isolates, and turkey-source-only isolates only, were reconstructed based on the alignments of core SNPs plus monomorphic sites with IQ-TREE (v1.6.10) (Nguyen et al., 2015). ModelFinder was used to identify the most appropriate substitution models according to the Bayesian information criterion (Kalyaanamoorthy et al., 2017). Branch support for both trees was estimated by performing 1,000 ultrafast bootstrap approximation replicates (Minh et al., 2013). The resulting trees were visualized and annotated using the online tool iTOL (Letunic and Bork, 2016). The all-isolate tree was annotated with metadata, including antibiotic resistance genes, CRISPR/Cas systems, and bacteriocins. Antibiotic resistance genes were identified using Abricate (https://github.com/tseemann/abricate) using the NCBI Bacterial Antimicrobial Resistance Reference Gene Database (Feldgarden et al., 2021). CRISPR/Cas systems were identified using CRISPRCasTyper (Russel et al., 2020). Bacteriocins were identified using BAGEL4 (van Heel et al., 2018).

Genome assemblies for each isolate were annotated with Prokka (Seemann, 2014), and core genome alignments were generated using Roary (v3.12.0) (Sitto and Battistuzzi, 2020) with 95% sequence identity. Scoary (v1.6.16) (Brynildsrud et al., 2016) was then used for pan-genome-wide association analysis first comparing chicken-source vs. turkey-source *L. johnsonii* isolates, then comparing clade A.4 vs. other turkey-source *L. johnsonii* isolates. Reference sequences of each significant gene were annotated using the top hit from a BLASTX search against the NCBI's nonredundant protein sequence database (Altschul et al., 1990).

### Bile Salt Hydrolase Activity

Bile salt hydrolase activity was assessed for turkey-source isolates (n = 85) by first inoculating 10 mL of MRS broth with 1 µL loopful of glycerol stock of each test strain, and incubating at 37ºC for 18 h (overnight) with shaking in aerobic conditions. MRS agar plates were prepared containing 0.5% (w/v) taurodeoxycholic acid sodium salt (MilliporeSigma, Burlington, MA). MRS agar plates without supplements were prepared as controls. Plates were inoculated via streaking with each test isolate, and incubated anaerobically at 41ºC for 48 h. Using colony morphology, bile salt hydrolase activity was identified for each isolate grown on taurodeoxycholic acid positive plates, following a previous protocol (Owusu-Kwarteng et al., 2015). Two replications were performed per isolate.

### Bile Salt Tolerance

For assessing bile salt tolerance, each turkey-source isolate (n = 85) was first grown in 10 mL of MRS broth at 37ºC for 18 h with shaking in aerobic conditions to reach 10^8^ CFU/mL. MRS broth containing 0.03% bile salts (Bile Salts, #3, Criterion) with the pH adjusted to 8.0 (using 1N NaOH) was added into a 96 well plate reader microtiter plate with 200 µL of media being added to each well. For matched controls, wells containing 200 µL of MRS broth without added bile salt, pH adjusted to 8.0 (using 1N NaOH), were included into the same plate. Then, 5 µL of overnight culture were inoculated into both bile and control wells. Representative wells containing both media types were left uninoculated for blank subtraction. Using an Epoch 2 plate reader (BioTek Instruments, Winooski, VT), plates were incubated for 18 h at 37ºC with shaking in aerobic conditions. OD_600_ measurements were taken every 10 min for the duration of the incubation. Blank subtraction (using Gen5 v3.08 software) and area under the curve were calculated for both bile positive and bile negative 18-h growth curves for each isolate. These experiments were run in replicates of 5 with 2 biological replications for a total of 10 replicates per isolate. A subset of 20 isolates were also tested at 0.15%, and 0.3% using the same methods used for 0.03% except with increased bile concentrations. These experiments were run in replicates of 2, with 2 biological replications, for a total of 4 replicates per isolate.

### Acid Tolerance

For acid tolerance, each turkey-source isolate (n = 85) of *L. johnsonii* was grown in 5 mL of MRS broth, at 37ºC for 18 h with shaking in aerobic conditions to reach 10^8^ CFU/mL. MRS broth was prepared with a pH adjusted to 2.0, 3.0, and 4.0 using 0.1N HCl, then filter sterilized with a 0.2 um filter. Then, 200 µL of each was added into a 96 well plate reader microtiter plate. For matched controls, 200 µL of MRS broth without pH adjustment (pH 6.0) were included on the same plate. Then, 5 µL of overnight culture was inoculated into pH 2.0, 3.0, 4.0, and 6.0 wells. Representative wells containing all 3 media types were left uninoculated for blank subtraction. Using an Epoch 2 plate reader, plates were incubated for 18 h at 37ºC degrees with shaking under aerobic conditions. OD_600_ measurements are taken every 10 min for the duration of the incubation. Blank subtraction (using Gen5 v3.08 software) and area under the curve were calculated for 18-h growth curves for each isolate and condition. Area under the curve percent differences between pH 2.0, 3.0, or 4.0 and pH 6.0 18-h growth curves were determined for each isolate. These experiments were run in replicates of 2, with 2 biological replications, for a total of 4 replicates per isolate.

### Avian Cell Association Assay

A subset of 21 turkey-source isolates were selected which were evenly distributed across the turkey-source phylogenetic tree, for further testing using an avian cell association assay. Budgerigar Abdominal Tumor Cells (**BATCs**) (Thomas et al., 2019) cells were grown in a complete medium of Dulbecco's modified Eagle medium (**DMEM**) (Thermo Fisher Scientific, Waltham, MA) supplemented with 20% Fetal Bovine Serum (Thermo Fisher Scientific, Waltham, MA). Cells were grown in 75 cm^2^ cell culture flasks (CellBIND surface; Corning Inc., Corning, NY) and incubated at 37ºC under 5% CO_2_. The cells were cultured to reach >95% confluence. The confluent cells were seeded into 24-well tissue culture plates (Corning Inc., Corning, NY) containing 1 mL complete medium and grown for 48 h, to reach >95% confluence (10^5^ cells/wells) in the wells. Each strain tested was first grown in MRS broth at 37ºC for 18 h with shaking in aerobic conditions to reach 10^8^ CFU/mL. These bacteria were centrifuged at 4,000 x *g* for 10 min at 25ºC, washed once, and resuspended in PBS. An initial OD_600_ reading was taken for each isolate prior to inoculation. BATC cells in the 24 wells (10^5^ cells/wells) were washed 3 times with PBS. The BATC cells immersed in 1 mL PBS were then inoculated with 10^6^ CFU/mL of each test strain separately and incubated for 2 h at 37ºC under 5% CO_2_. Following incubation, the cells were washed 3 times with PBS to remove the unattached bacterial cells. Cells were then incubated in 1 mL of 0.1% Triton X-100 for 15 min at 37ºC under CO_2_ for host cell lysis. Cell lysates were then serially diluted in PBS, and appropriate dilutions of lysates were plated on MRS plates and incubated for 48 h at 37ºC in anaerobic conditions. Plates were counted for CFU/mL determination. To calculate the initial CFU/mL for each isolate the individual OD_600_ reading was inserted into to a calibration curve equation determined for *L. johnsonii* which was: CFU/mL initial = ((10^8^*OD_600_) - (5*10^6^)). Percent adherence was calculated by dividing the final CFU/mL by the initial CFU/mL and multiplying by 100 for each replicate.

### Turkey Performance Trial

All animal work was reviewed and approved by the University of Minnesota Institutional Animal Care and Use Committee under protocol 1809-36339A. To assess the ability of turkey-source *L. johnsonii* to impact early turkey poult performance, cage trials were conducted. For this trial, Hybrid males were purchased from a commercial source (Select Genetics, Willmar, MN). There were a total of 15 experimental cages, with 8 control replicates and 7 UMNLJ21-inoculated replicates. Each experimental unit (cage) contained 10 turkey poults. These were run across 2 trials. In trial 1, 5 different isolates representing the 5 turkey-source phylogenetic clades were individually tested. In the second trial, 1 isolate belonging to one of the clades was repeated. At d 0 of age, the inoculated group was administered an oral gavage of 0.5 mL (PBS) containing 8.0 × 10^8^ colony forming units of the targeted probiotic strain. Birds were fed ad libitum with an industry-standard starter diet. Lighting and temperature control followed hybrid recommendations. At d of hatch, birds were sorted by weight, distributed across pens, and placed on fresh litter shavings. Individual and pen-level weights were obtained at d 0, 7, and 14. Feed was also weighed back at these time points to calculate feed intake per bird, per d.

### Broiler Performance Trial

To assess the ability of host-adapted *L. johnsonii* to impact broiler performance, a pen trial was conducted. For this trial, Ross 708 broiler chicks were purchased from a commercial source (Welp Hatchery, Bancroft, Iowa). There were a total of 48 experimental pens used, with 12 replicates each of 4 different treatment groups. Each experimental unit (pen) contained 13 broiler chicks. At d 0 and 7 of age, groups were administered an oral gavage of (PBS) (Control group) or PBS containing 8.0×10^8^ colony forming units of the targeted probiotic strain. Birds were fed ad libitum with an industry-standard 3-phase mash broiler diet. Diet changes occurred at d 14 and 28 of age. At d of hatch, birds were sorted by weight, distributed across pens, and placed on fresh litter shavings. Feed intake per pen was measured weekly. For bird weights, feed was withheld for 6 h prior to weighing, and individual body weights were recorded weekly.

### Statistical Analyses

For pangenomic comparisons, a gene was reported as significantly associated with a comparison group if it had a Benjamini-Hochberg (**BH**)-adjusted *P* ≤ 0.05, and was present in ≥50% of isolates in the group of interest and ≤50% of isolates from the other comparator group. Area under the curve percent differences between bile positive and bile negative 18-h growth curves were determined for each isolate (Sprouffske and Wagner, 2016). Percent difference in area under the curve between bile positive and bile negative growth curves were compared between clades using a 1-way ANOVA (randomized design). For acid tolerance assays, percent difference in area under the curve between the pH-adjusted (pH 2.0, 3.0, 4.0) and non-pH-adjusted (pH 6.0) growth curves were compared between clades using a 1-way ANOVA (randomized design). For BATC association assays, clade differences were calculated using a 1-way ANOVA (randomized design). Post hoc comparisons were performed for all comparisons using Tukey's test. All tests were calculated with R v.4.0.3. For bird performance trials, statistics were conducted in SAS Studio version 3.8 using the PROC MIXED function, with *P* ≤ 0.1 as the level of significance.

### Data Deposition

Raw reads from isolates sequenced in this study are available at the NCBI short read archive (**SRA**) under BioProject accession no. PRJNA316010. The completed genomes of strain UMNLJ21 is available through NCBI under accession numbers CP021703 (chromosome) and CP021701- CP021702 (plasmids).

## RESULTS

### Isolation of Turkey-Source *L. johnsonii*

Based upon previous data indicating a positive correlation between *L. johnsonii* and weight gain in commercial turkeys, we focused on the isolation of *L. johnsonii* for assessment of its potential as a DFM in poultry. From historically high-performing turkey flocks spanning 1 to 10 wk of age, a total of 1,268 isolates were obtained. Of these, 197 isolates had best-hit BLAST matches (>95% similarity) to *L. johnsonii* using 16S rRNA sequencing. After further screening with *L. johnsonii*-specific primers, 118 turkey *L. johnsonii* isolates were confirmed for subsequent DNA sequencing and analyses. In addition to *L. johnsonii*, a variety of other species were identified from these samples, including *L. crispatus, L. helveticus, Limosilactobacillus reuteri, Ligilactobacillus salivarius, Ligilactobacillus aviarius, Limosilactobacillus vaginalis, Pediococcus acidilactici,* and *Enterococcus hirae*.

**Figure 1:**
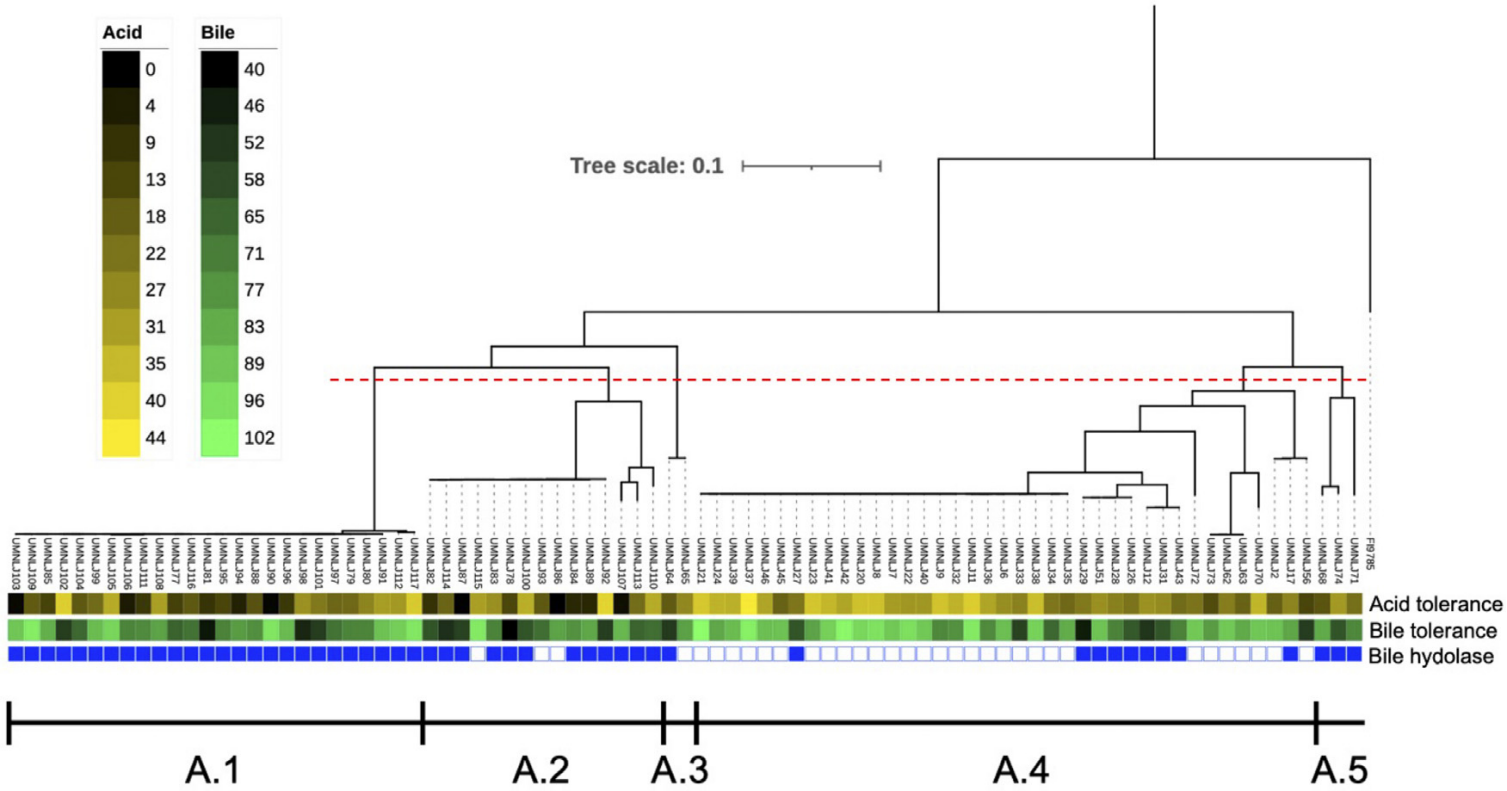
Phylogenetic tree depicting 85 turkey-source *L. johnsonii* isolates, designated clade A. Isolates are grouped in subclades A.1 through A.5 based upon a subjective cut-off (red dashed line). Chicken-source strain FI9785 is included as an outgroup. Heatmaps depict percent tolerance to acid (pH 2.0) and bile salts (0.03%). Blue bar at bottom shows bile salt hydrolase activity (blue) or no activity (white).

Following freezing of these isolates and storage at -80ºC using standard conditions, 85 out of 118 turkey-source *L. johnsonii* isolates were readily recovered and performed favorably in standard growth conditions. Therefore, these 85 isolates were used for in vitro assays of probiotic potential. First, a phylogenetic tree was constructed for the 85 genomes using a core SNP-based maximum likelihood analysis (Figure 1). This was designated “clade A,” and was further subdivided into 5 subclades (A.1 through A.5) for comparison purposes. Of note, strain UMNLJ21, which has been previously characterized (Ward et al., 2019), belonged to subclade A.4.

### Assays for Probiotic Potential Identify Differences by Clade

**Figure 2:**
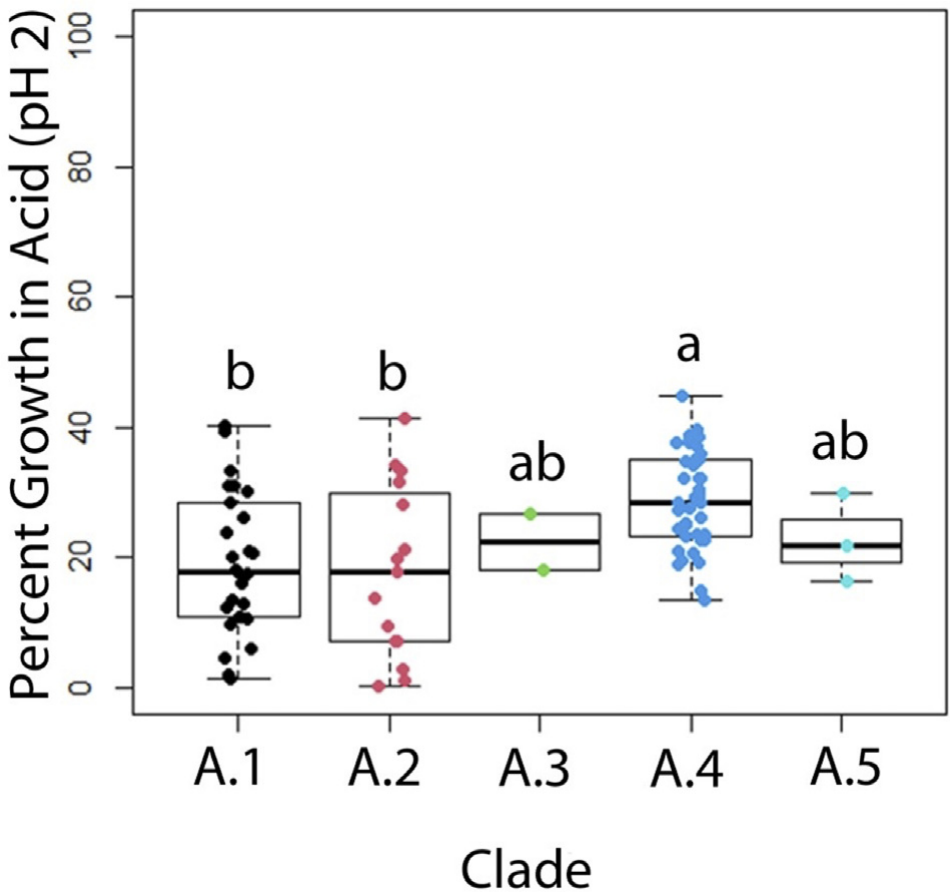
Percent area under the curve growth in MRS broth adjusted to a pH of 2 by *L. johnsonii* subclade (n = 85). Each point represents the mean result of 4 replicates of an individual isolate. Statistical significance is represented as *p* ≤ 0.05. Data on other concentrations tested are included in the supplementary material.

**Figure 3:**
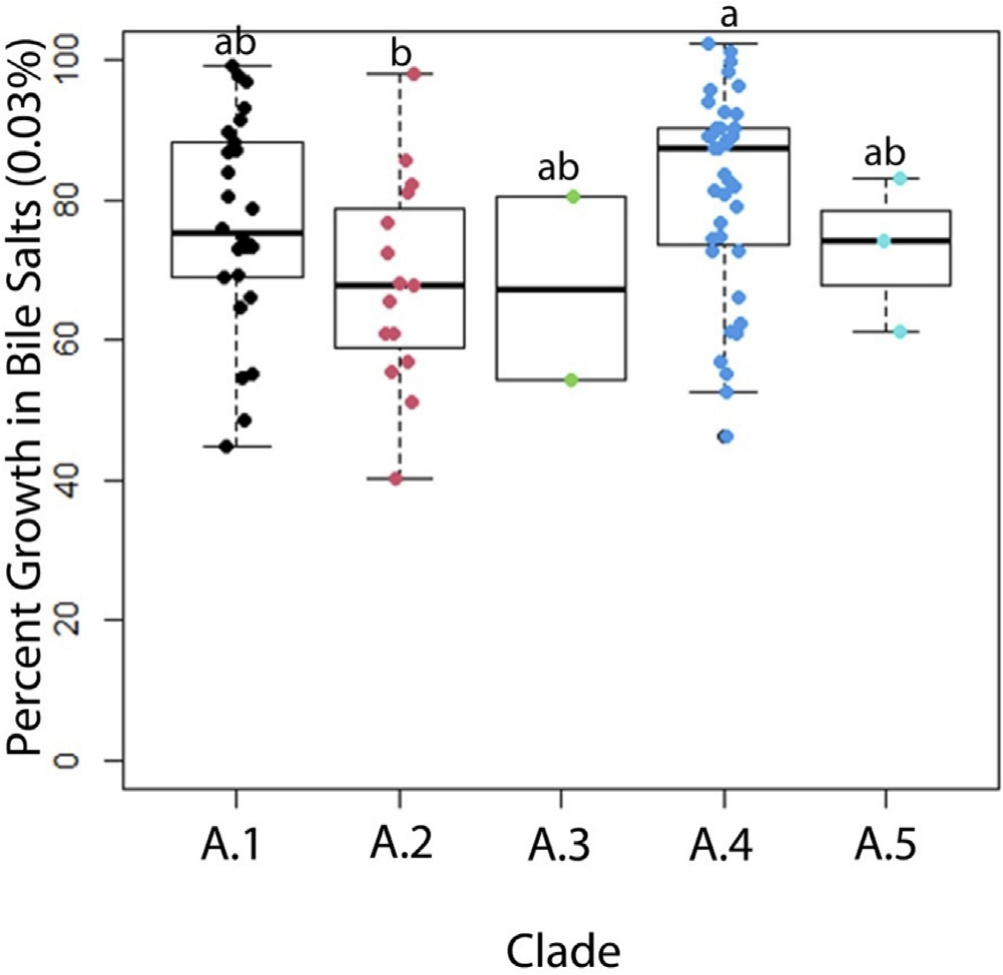
Percent area under the curve growth in MRS broth containing 0.03% w/v bile salts by *L. johnsonii* subclade (n = 85). Each point represents the mean result of 10 replicates of an individual isolate. *p* ≤ 0.05. Data on other concentrations tested are included in the supplementary material.

In vitro assays for acid tolerance, bile salt tolerance, and bile salt hydrolase activity were performed for all 85 turkey-source *L. johnsonii* isolates. At pH 2.0, isolates from subclade A.4 performed significantly better (*P* < 0.05) than subclades A.1 and A.2, and numerically better than subclades A.3 and A.5 (Figure 2). Isolates were tested in media containing bile salts at a concentration of 0.03%. A subset of 20 isolates were also tested at 0.15%, and 0.3%. At 0.03% bile salts, isolates from subclade A.4 performed significantly better than subclade A.2, and numerically better than all other clades (Figure 3). Similar results were obtained at 0.15% and 0.3% bile salts, although strains in general grew less efficiently with increasing percentages of bile salts (Supplementary Data). A simple bile salt hydrolase assay was also performed on all 85 isolates. In this case, all isolates from subclades A.1 and A.5 were bile salt hydrolase positive, whereas there was a mix of positive and negative isolates within other subclades. In subclade A.4, interestingly, 77% of isolates were bile salt hydrolase negative, in contrast to this clade performing most efficiently in the presence of mixed bile salts in liquid media.

A subset of 21 out of the 85 turkey-source isolates, representing the different clades in the phylogenetic tree, were further tested for their ability to associate with BATC cells as a model of ability to adhere within the avian gastrointestinal tract. In this assay, no significant differences were observed between subclades (Supplementary Data), with isolates associating with BATC cells at frequencies of 72 to 89%.

Due to their increased tolerance to acid and greater ability to grow in the presence of bile salts, it was concluded that strains from subclade A.4 exhibited the greatest potential as probiotic candidates. The strain ultimately chosen from this subclade was bile hydrolase negative; however, because it performed quite favorably in mixed bile salts vs. only taurodeoxycholic acid in the bile hydrolase assay, and this strain had been previously characterized, it was used for subsequent in vivo experiments.

### A Single Inoculation of a Strain From Subclade A.4 Enhances Early Turkey Poult Performance

Following identification of subclade A.4 as having the greatest probiotic potential among turkey-source *L. johnsonii*, two 14-d caged trials were conducted in turkey poults to determine if oral application of a subclade A.4 strain at hatch had a positive impact on early poult performance.

In the first trial, representative strains from each of subclades A.1 to A.5 were used and compared to a negative control group. Poults from the subclade A.4-inoculated group had significantly higher average body weights than all other groups at d 14 (*P* < 0.1), with a difference of +39.9 g or +12.2% compared to the negative control group (Table 1). Similarly, average daily gain from d 0 to 14 was significantly higher for the subclade A.4-inoculated group compared to all other groups (Table 1). In contrast, no significant differences across groups were observed for feed efficiency or average daily feed intake (Table 1).

**Table 1:** Effects of turkey-source *L. johnsonii* administration on early poult performance.

	D 0 body weight (g)	D 7 body weight (g)	D 14 body weight (g)	D 7 CV	D 14 CV	D 0–14 average daily gain (g)	D 0–14 average daily feed intake (g)	D 0–14 FCR
Control	59.2 ± 0.5	141.1 ± 4.1	326.6 ± 2.9^b^	11.2 ± 1.4	10.6 ± 2.8	19.1 ± 0.2^b^	20.9 ± 1.6	1.39 ± 0.09
Subclade A.1	59.9 ± 0.7	143.2 ± 2.6	324.0 ± 6.1^b^	7.9 ± 1.1	4.5 ± 1.6	18.9 ± 0.4^b^	20.6 ± 1.0	1.41 ± 0.09
Subclade A.2	56.7 ± 1.5	135.5 ± 1.2	318.6 ± 11.7^b^	15.7 ± 4.0	19.7 ± 5.1	18.7 ± 0.7^b^	21.6 ± 0.6	1.45 ± 0.02
Subclade A.3	59.3 ± 0.5	145.5 ± 3.1	334.7 ± 2.2^b^	9.4 ± 2.7	9.0 ± 2.3	19.8 ± 0.2^b^	21.7 ± 1.0	1.38 ± 0.08
Subclade A.4	57.7 ± 0.1	148.1 ± 4.9	366.5 ± 15.4^a^	10.4 ± 1.9	9.6 ± 2.0	21.9 ± 1.0^a^	23.6 ± 1.0	1.41 ± 0.03
Subclade A.5	57.1 ± 1.8	143.3 ± 3.9	333.5 ± 4.5^b^	8.0 ± 3.1	7.7 ± 1.9	19.7 ± 0.4^b^	21.3 ± 0.5	1.40 ± 0.08
*P* value	0.23	0.27	0.02	0.35	0.05	0.02	0.40	0.99

Abbreviations: CV, coefficient of variation; FCR, feed conversion ratio.

a,b Superscript letters indicate *P* < 0.05.

A second trial was conducted using only a noninoculated control, compared to poults from a subclade A.4-inoculated group. Combining the 2 trials together, the subclade A.4-inoculated group had significantly higher average body weights compared to control at d 14 (*P* < 0.1), with a difference of +22.6 g or +6.7% (Table 2).

**Table 2:** Effects of turkey-source subclade A.4 *L. johnsonii* administration on early poult performance, across 2 trials.

	D 0 body weight (g)	D 7 body weight (g)	D14 body weight (g)	D 7 CV	D 14 CV	D 0–14 average daily gain (g)	D 0–14 average daily feed intake (g)	D 0–14 FCR
Control (trial 1)	59.2 ± 0.5	141.1 ± 4.1	326.6 ± 2.9	11.2 ± 1.4	10.6 ± 2.8	19.1 ± 0.2	20.9 ± 1.6	1.393 ± 0.087
Subclade A.4 (trial 1)	57.7 ± 0.1	148.1 ± 4.9	366.5 ± 15.4	10.4 ± 1.9	9.6 ± 2.0	21.9 ± 1.0	23.6 ± 1.0	1.407 ± 0.034
Control (trial 2)	65.3 ± 0.2	148.1 ± 3.9	349.7 ± 11.3	10.4 ± 2.6	11.0 ± 5.2	20.6 ± 0.9	19.6 ± 0.6	1.350 ± 0.043
Subclade A.4 (trial 2)	66.3 ± 0.1	149.3 ± 3.4	360.0 ± 6.5	7.5 ± 1.8	6.2 ± 2.4	20.7 ± 0.6	19.6 ± 0.5	1.312 ± 0.011
Control (trials combined)	62.7 ± 1.3	145.1 ± 3.0	339.8 ± 7.7	10.7 ± 1.5	10.8 ± 3.0	19.9 ± 0.6	20.2 ± 0.7	1.369 ± 0.041
Subclade A.4 (trials combined)	63.1 ± 1.6	148.8 ± 2.6	362.4 ± 6.5	8.6 ± 1.3	7.7 ± 1.7	21.2 ± 0.5	21.1 ± 0.9	1.348 ± 0.022
*P* value (trials combined)	0.87	0.36	0.04	0.31	0.38	0.13	0.44	0.65

### Subclade A.4 Isolates Possess a Subset of Genomic Traits Absent From Other *L. johnsonii* Clades

**Figure 4:**
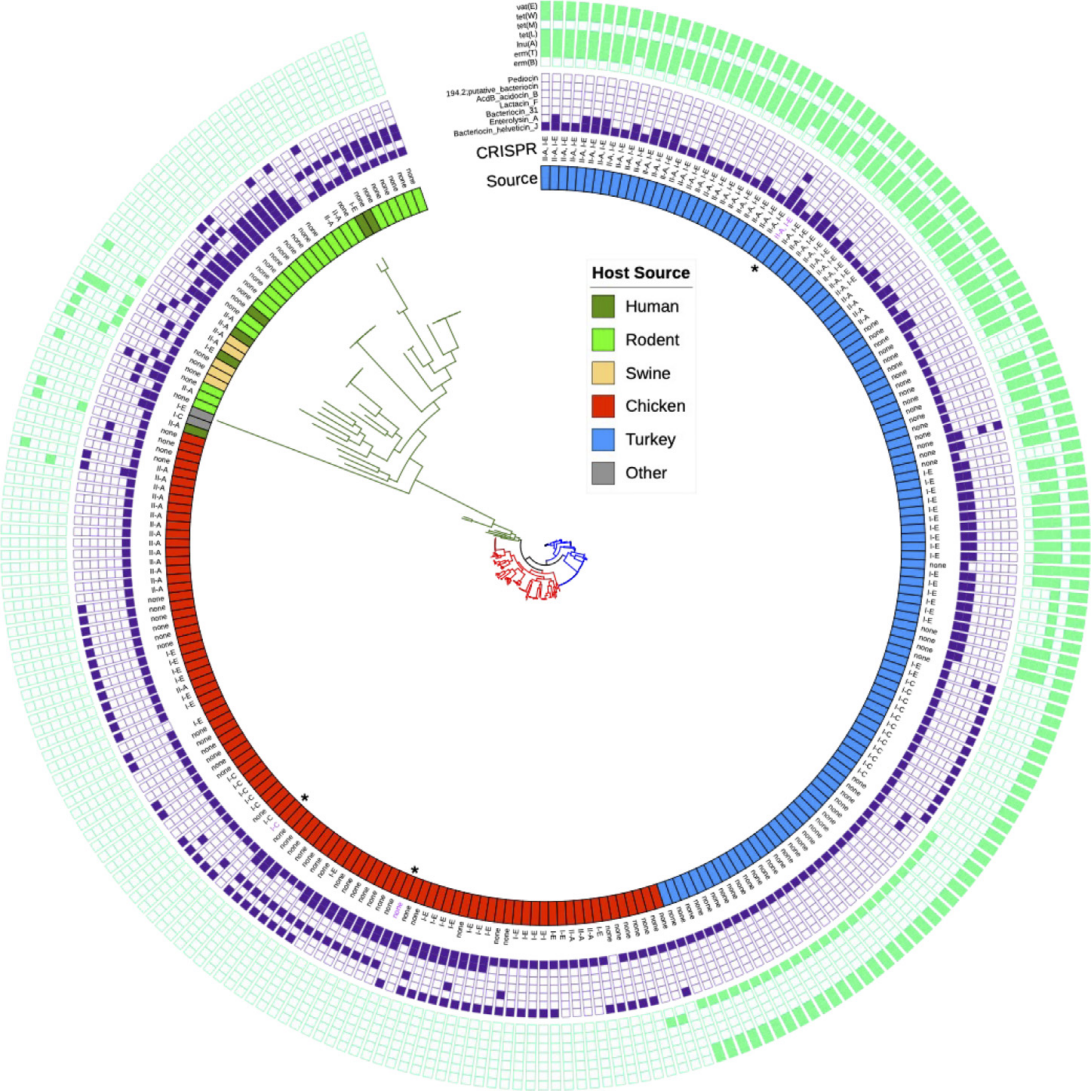
Maximum likelihood phylogenetic tree based upon core SNPs within *L. johnsonii*. A total of 57,166 SNPs is represented in the dataset. The outer seven rings (light green) depict presence of antibiotic resistance genes. The middle seven rings (purple) depict presence of bacteriocins. The inner 2 rings depict host course and CRISPR/Cas system presence. Isolates with asterisks inside the Source ring are those used individually for subsequent in vivo trials. Tree branches are colored to designate clade A (blue), clade B (red), and clade C (green).

Using pangenomic comparisons between all subclade A.4 and all other *L. johnsonii* turkey-source subclades, a total of 221 genes were identified that were present in subclade A.4 genomes at >50% prevalence but were significantly less prevalent in other clades (<50% prevalence; *P* < 0.05). These genes were then mapped back to the completed chromosome and plasmids of strain UMNLJ21. A large proportion of the genes identified (69.7%) were classified as hypothetical proteins. Also identified as subclade A.4-specific were seven genes classified as CRISPR/Cas-associated, which is in line with the identification of 2 CRISPR/Cas regions in subclade A.4 belonging to types II-A and I-E, with I-E being over-represented in subclade A.4 isolates (Figure 4). These 2 CRISPR/Cas systems were found to be colocated in the genome of strain UMNLJ21, separated by approximately 10 kbp. Additional genomic islands identified among subclade A.4 isolates included a putative restriction-modification system, putative sugar utilization and transport systems of unknown specificity, and associated insertion sequence/transposase elements that have likely facilitated integrations of these regions.

Using the completed sequence of a representative subclade A.4 strain (UMNLJ21), a putative integrative conjugative element (**ICE**) was identified in its genome that corresponded with some of the subclade A.4-specific genes identified using pangenomic approaches. This ICE encompassed a 46,951-bp region from sites 1,421,641 to 1,468,591 in the UMNLJ21 chromosome. This region contained direct repeats and predicted integrase, relaxase, and other type IV secretion system components. Most of the subclade A.4-specific genes identified within this putative ICE were core components of the ICE itself, and some hypothetical proteins. Also identified within this region was a predicted class III bacteriocin, MFS transport system, and ABC transport system. This ICE also contained genes which were 99% similar to *bcrABDR* on *Clostridium perfringens* plasmid pJIR4150, previously shown to encode for bacitracin resistance (Han et al., 2015).

### *L. johnsonii* Exhibits Phylogenetic Signals of Coadaptation With its Host

While the coadaptation of *L. johnsonii* with its host has been proposed (Guinane et al., 2011), this has been done so only using limited genomic comparisons. Furthermore, it was unclear if such coadaptation extends to closely related animal hosts, such as chickens and turkeys. Thus, we sought to isolate *L. johnsonii* from commercial broilers, using a species-specific PCR screen to initially identify isolates. Four hundred total isolates were obtained from multiple commercial broiler flocks and screened following isolation on MRS or LBS agar; 86 of these isolates were confirmed to be *L. johnsonii*. Non-*L. johnsonii* isolates from broilers were not further characterized.

All *L. johnsonii* isolates sequenced in this study (n = 204, 118 turkey-source and 86 chicken-source) plus additional database-deposited isolates were then subjected to phylogenetic analyses, based upon core genomic SNPs (Figure 4). A total of 57,166 informative core SNPs were identified in the collective dataset. Trees were constructed with and without removal of recombinant regions using Gubbins. In both cases, the inferred trees illustrated clustering of isolates by host source (data not shown). These clades were designated clade A (turkey-source isolates), clade B (chicken-source isolates), and clade C (mammalian-source isolates). There was a clear separation of clades not only between mammalian-host vs. avian-host isolates, but also between chicken-host vs. turkey-host isolates. These clusters included not only isolates collected in this study, but also additional chicken-source isolates collected within and outside of North America. An analysis of core SNP distances between the different groups of isolates further supported the concept of coadaptation between *L. johnsonii* and its host (Table 3). While within-group mean SNP distances were 1,690 and 1,993 for turkey and chicken isolates, respectively, the mean SNP distance between chicken and turkey isolates was 2,447. Furthermore, mean SNP distance between avian and mammalian isolates was greater than 10,000 SNPs, while the within-mammal mean SNP distance was 6,572. Collectively, this supports the idea of coevolution of host and *L. johnsonii,* a phenomenon which has been previously reported for lactobacilli (Duar et al., 2017).

**Table 3:** Core genome SNP differences between isolates, categorized by host source.

Source comparison	Mean SNPs	SD	Minimum difference	Maximum difference
Turkey vs. turkey	1,690	719	0	2,436
Chicken vs. chicken	1,993	1,242	0	9,853
Chicken vs. turkey	2,447	778	1943	9,877
Mammal vs. mammal	6,572	2,212	0	12,896
Chicken vs. mammal	10,132	1,205	4,259	13,188
Turkey vs. mammal	10,141	1,161	4,294	13,264

Minimum and maximum differences refer to minimum and maximum SNP differences among strains between comparator groups.

Using pangenomic comparisons, a total of 172 genes were identified that were present in turkey-source *L. johnsonii* genomes at >50% prevalence, but were significantly less prevalent in chicken-source *L. johnsonii* clades (<50% prevalence; *p* < 0.05). A putative streptogramin resistance gene *vat(E)* was identified in all turkey-source isolates, but no chicken-source isolates. Using the representative subclade A.4 completed genome, this gene was found on a genomic island, bound by IS*30*-like elements, and adjacent to a restriction-modification system which was also specific to turkey-source isolates. A tetracycline resistance gene *tet(W)* was present in nearly all turkey-source isolates but absent from all chicken-source isolates. This gene was also located in the representative subclade A.4 completed genome, but its apparent insertion mechanism was unclear because it was not adjacent to any transposable elements and did not appear to be located on a genomic island.

A second putative ICE was identified in the representative subclade A.4 genome that corresponded with some of these turkey-specific genes identified using pangenomic approaches. This was a 45,276-bp region from sites 635,369-654,369 in the UMNLJ21 chromosome, and shared highest database similarity with an ICE from *L. lactis* strain KF147 (Siezen, et al., 2010). This region contained direct repeats and predicted integrase, relaxase, and other type IV secretion system components. Carried within this putative ICE were genes predicted to confer beta-galactosidase activity, *lnu(A)* predicted to confer lincosamide resistance, and *tet(L)* predicted to confer tetracycline resistance.

Because of the identification of several antimicrobial resistance genes within poultry-source *L. johnsonii* strains, we examined all strains for their possession of acquired resistance genes using the NCBI's AMRFinderPlus tool. Sharp differences were observed between turkey-source and chicken-source isolates. The chicken-source isolates were almost completely devoid of acquired resistance genes. In contrast, 100% of the turkey-source isolates examined contained *vat(E)* and *tet(W)*, and >50% of them contained *tet(L), lnu(A)*, and *erm(T)* (conferring erythromycin resistance). Patterns of resistance gene carriage were observed by subclade (Figure 4). Clade A.1 carried a unique pattern of *vat(E), tet(W)*, and *erm(B)*; whereas clade A.4 isolates carried a pattern of *vat(E), tet(W), tet(L), lnu(A)*, and *erm(T)*.

We also examined the presence of CRISPR/Cas systems and bacteriocins among all isolates. In chicken-source isolates, 8% of isolates harbored a type I-C CRISPR/Cas system, 26% harbored type I-E, and 18% harbored type II-A. In turkey-source isolates, 10% carried type I-C, 45% carried type I-E, and 33% carried type II-A. Of those, 29% of turkey isolates carried both types II-A and I-E, while none of the chicken isolates had this combination. Nearly all isolates in this study carried a bacteriocin classified as helviticin J. Chicken-source isolates contained a gene classified as enterolysin A at a frequency of 26%, compared to 35% of turkey-source isolates harboring this gene. Other predicted bacteriocins found among avian isolates included acidocin B (found in 26% of chicken-source isolates and 4% of turkey-source isolates), and pediocin (60% of chicken-source isolates and 13% of turkey-source isolates).

### Chicken-Adapted *L. johnsonii*, but not Turkey-Adapted *L. johnsonii*, Enhance Broiler Performance Over 42 D

We next sought to determine if host-adapted strains from chickens and turkeys exhibit differences in their ability to impact performance, using a broiler pen trial. Broilers were chosen because of their relatively faster time to market age, and the ability to achieve higher replicates using this model. In this experiment, 2 broiler strains of *L. johnsonii* were selected based on phylogeny (belonging to different clade B subclades and differing by core 1,953 SNPs) and phenotypic data confirming similar acid tolerance (27.4% and 26.1%, respectively), bile salt tolerance (95.4% and 85.5%, respectively), and BATC association (73.9% and 80.5%, respectively) to turkey-source clade A.4 isolates. These groups were then individually compared to a noninoculated control group, and a UMNLJ21-inoculated group (turkey-source *L. johnsonii*). In this study, oral gavages were administered to each bird in the group at d 0 and 7 of age. The reasoning for this approach was to determine if application of host-adapted probiotics during the first week of age, rather than continuously, are capable of enhancing performance through market age. Also, if strain host adaptation plays a role in performance enhancement, we expected that benefits from a non-host-adapted probiotic would erode over time.

For body weights, significant differences (*P* = 0.1) were observed at d 7 of age, with chicken-source groups and turkey-source group having numerically higher average body weights compared to the control group, ranging from +4.2% to +5.4% (Table 4). At a market age of 42 d, the chicken source *L. johnsonii* groups averaged +3.1% and +2.4% heavier body weights than the control group, respectively, and the turkey-source group was only +0.8% compared to the control. For FCR, significant improvements (*P* < 0.1) of 3 FCR points were observed from d 0 to 7 for the turkey-source and chicken-source #2 probiotic, compared to the control group (Table 5). No other significant differences in FCR were observed for other time intervals. No significant differences were observed in the trial for feed consumption or mortality (Supplementary Data).

**Table 4:** Body weights of broiler chickens administered host-specific *L. johnsonii*.

	D 0 body weight (g)	D 7 body weight (g)	D 14 body weight (g)	D 28 body weight (g)	D 35 body weight (g)	D 42 body weight (g)
Control	35.9 ± 0.0	168^b^ ± 2	479 ± 5	1,718 ± 15	2,530 ± 17	3,281 ± 31
Turkey UMNLJ21 probiotic	35.9 ± 0.0	176^a^ ± 2	487 ± 6	1726 ± 31	2,509 ± 45	3,307 ± 38
Chicken LJ probiotic #1	35.9 ± 0.0	175^a^ ± 3	490 ± 5	1,763 ± 15	2,584 ± 16	3,382 ± 21
Chicken LJ probiotic #2	35.9 ± 0.0	177^a^ ± 3	494 ± 5	1,761 ± 14	2,564 ± 22	3,360 ± 36
*P* value	0.51	0.10	0.26	0.28	0.23	0.13

a,b Superscript letters indicate statistically significant differences (*P* ≤ 0.1).

**Table 5:** Feed conversion ratios of broiler chickens administered host-specific *L. johnsonii*.

	D 0–7 FCR	D 0–14 FCR	D 0–28 FCR	D 0–35 FCR	D 0–42 FCR
Control	1.01 ± 0.01^a^	1.12 ± 0.00	1.27 ± 0.00	1.37 ± 0.01	1.48 ± 0.01
Turkey UMNLJ21 probiotic	0.98 ± 0.01^b^	1.12 ± 0.00	1.27 ± 0.01	1.37 ± 0.01	1.48 ± 0.01
Chicken LJ probiotic #1	1.00 ± 0.01^a^^,^^b^	1.12 ± 0.01	1.28 ± 0.01	1.37 ± 0.01	1.46 ± 0.01
Chicken LJ probiotic #2	0.98 ± 0.01^b^	1.12 ± 0.01	1.27 ± 0.01	1.37 ± 0.01	1.47 ± 0.01
*P* value	0.03	0.89	0.87	0.99	0.65

a,b Superscript letters indicate statistically significant differences (*P* < 0.05).

## DISCUSSION

Direct-fed microbials, or probiotics, are frequently used in poultry production with goals of inhibiting pathogens, improving performance, and enhancing gut health (Abd El-Hack et al., 2020). In the current commercial landscape of probiotics for poultry, the vast majority are applied continuously in feed. Because of the requirements for viability in poultry feed, these organisms either need to be sporulated or they need to be encapsulated. Fewer vegetative microorganisms are available as probiotics compared to spore-forming bacteria, and these products often require that they be administered at hatch or via water in an established flock (Abd El-Hack et al., 2020).

The purpose of this study was to explore the idea that host-specific probiotics can be identified through a top-down approach, and that they can be administered infrequently and still have a long-term positive impact on performance. This also afforded limited opportunity to explore the concept of host adaptation as it applies to *L. johnsonii*. Lactobacilli have been referred to as a driving force in the evolution of an animal's immune system, as they appear to coevolve with their host while retaining an interaction that avoids excessive immune reaction (O'Callaghan and O'Toole, 2013). The results of this study confirm that, genetically, *L. johnsonii* clearly cluster phylogenetically by host source. This extends even to relatively closely related host species, such as chickens (*Gallus gallus domesticus*) and turkeys (*Meleagris gallopavo domesticus*).

Phenotypic assays for probiotic potential are routinely used in research and development towards commercialization (Suez et al., 2019). One aspect of probiotic efficacy, which is difficult to model in the laboratory, is how the probiotic will behave once it has transited to the lower gastrointestinal tract. Some of the in vitro models used here attempted to reproduce key conditions required for survival during gastrointestinal transit, including exposure to acidic pH and to bile salts in the upper small intestine. Differences were observed in these assays and indicated that subclade A.4 turkey-source isolates and selected chicken-source isolates may survive transit better than isolates from other *L. johnsonii* subclades. In contrast, our single in vitro model of behavior once the probiotic strain has transited to the lower gastrointestinal tract (association to BATC cells) showed no significant differences between strains of different clades. However, the gut is complex, and these models by design are simplistic and artificial. Furthermore, use of BATC (which are avian cells but are not derived from chickens or turkeys) was convenient, but host-derived primary cells or cell lines may be important to adequately assess colonization capability (Cencic and Langerholc, 2010). Alternatively, a more complex in vitro system which includes multiple cell types and gut conditions may be required. Nonetheless, there was utility in our screening approach, in that it was able to filter a larger collection of isolates down to a subset of candidates based on phenotypic characteristics believed to be associated with better probiotic performance in vivo.

In vitro systems lack the capacity to reproduce conditions across the entire gastrointestinal tract, and more so, they are unable to predict how a probiotic will behave once in the actual target host (Cencic and Langerholc, 2010). However, testing with sufficient power and replication to discern differences in poultry performance is costly and preventative towards testing large numbers of candidate isolates. Ideally, this work would have included additional pen replicates in the broiler trial, and a longer timeframe in the 2 turkey trials. However, we contend that intermediate screens in birds such as the early poult performance model used here can be quite useful for filtering out candidates for further study.

During selection of a wild type strain for probiotic use, it is important to consider risk of applying said strain to human and animal health. One aspect of risk is the carriage, or potential for acquisition, of antimicrobial resistance genes. While this was not an initial goal of this study, retrospective genomic comparisons revealed that all turkey-source *L. johnsonii* strains harbor fairly high numbers of resistance-encoding elements. In contrast, it was quite easy to identify broiler-source strains which lacked known resistance genes, since nearly all of them were absent of these genes. This did not appear to have an impact on strain performance, and this was expected since no antimicrobials were used in this study. Still, as probiotic candidates are identified, it is important to not only assess their current antibiotic resistance gene content, but also their propensity to mutate towards antibiotic resistance.

We do not know what implications, if any, the presence of CRISPR/Cas systems or bacteriocins may have on candidate strain performance. CRISPR/Cas systems are thought to be bacterial adaptive immune systems which can acquire cassette elements that target invading genetic material, such as that brought in by bacteriophage (Hille et al., 2018). There is evidence in lactobacilli that there may be an antagonistic association between the presence of CRISPR/Cas systems and genomic prophage elements (Pei et al., 2021). While we did not look at prophage in this study, nor did we examine CRISPR/Cas cassette content, this aspect of CRISPR/Cas would be important when considering the identification of probiotic candidates, as active phage could interfere with fermentation processes and could influence the avian microbiome in unexpected ways. With respect to bacteriocins, certainly it is well known that the production of these proteins by lactobacilli often enables competitive exclusion against other bacterial species. Helveticin J, which was found in nearly all isolates examined in this study, was first identified in *L. helveticus* (Joerger and Klaenhammer, 1986), and is a large molecular weight bacteriocin with a narrow spectrum of inhibitory activity within Lactobacillaceae. Enterolysin A is a cell wall-degrading bacteriocin which was first identified in *E. faecalis* (Nilsen et al., 2003), and genes with low sequence similarity to it have been found in lactobacilli that have inhibitory properties towards other microorganisms (Dos Santos et al., 2021). Pediocins have been identified in many lactobacilli and appear to have a broader spectrum of inhibitory activity which includes *Listeria, Enterococcus*, and *Clostridium* (Grilli et al., 2009; Balandin et al., 2019). While the inhibition of pathogens was not a focus of this study, the identification of naturally occurring probiotic candidates with complementary sets of bacteriocins is likely an important consideration when considering said candidates.

There were limitations to this study. We lacked sufficient statistical power to achieve significant differences in body weight, and possibly FCR, at all timepoints in each bird trial. Replication of this study with larger numbers of pen replicates would be ideal, both to achieve better statistical power and to provide additional biological replication. Also, it would have been ideal to conduct both broiler and turkey pen trials, each using both chicken- and turkey-adapted *L. johnsonii* isolates. Still, the combination of strong genomic and in vitro data with compelling (yet sometimes insignificant) in vivo data suggests that coadaptation of *L. johnsonii* does exist between poultry host sources, and that host-specific *L. johnsonii* may confer a stronger and longer lasting effect than non-host-specific strains.

This work demonstrates that top-down approaches such as this towards identifying host-adapted lactobacilli hold promise. Such strains have the potential to be applied during limited periods of the bird's growth cycle, such as at hatch and during stress periods. Future work on host-adapted strains should examine their impact on the gut microbiome, gut development, and interactions with the host immune system.

## Appendix Supplementary materials

Image, application 1

## Footnotes

Supplementary material associated with this article can be found, in the online version, at doi:10.1016/j.psj.2023.102858.
